# Altered Structural Brain Network Topology in Patients With Primary Craniocervical Dystonia

**DOI:** 10.3389/fneur.2022.763305

**Published:** 2022-03-30

**Authors:** Xiu Wang, Wenhan Hu, Huimin Wang, Dongmei Gao, Yuye Liu, Xin Zhang, Yin Jiang, Jiajie Mo, Fangang Meng, Kai Zhang, Jian-guo Zhang

**Affiliations:** ^1^Department of Neurosurgery, Beijing Tian Tan Hospital, Capital Medical University, Beijing, China; ^2^Beijing Key Laboratory of Neurostimulation, Beijing, China; ^3^Stereotactic and Functional Neurosurgery Laboratory, Beijing Neurosurgical Institute, Capital Medical University, Beijing, China; ^4^Department of Functional Neurosurgery, Medical Alliance of Beijing Tian Tan Hospital, Peking University First Hospital Fengtai Hospital, Beijing, China

**Keywords:** primary craniocervical dystonia, morphological connectivity, similarity, network connectivity, structural MRI

## Abstract

**Purpose:**

Regional cortical thickness or volume analyses based upon structural MRI scans have been employed to study the pathophysiology of primary craniocervical dystonia (CCD). In the present study, brain connectivity network analyses based upon morphological distribution similarities among different brain areas were used to study the network disruption in individuals affected by CCD.

**Methods:**

The T1 MRI scans were completed for 37 patients with CCD and 30 healthy controls, with individual brain structural networks being constructed based upon gray matter (GM) similarities in 90 regions within the brain. Area under the curve (AUC) values for each network parameter were determined, and the GRETNA program was used to conduct a graph theory-based measurement of nodal and global network properties. These properties were then compared between healthy controls and those with CCD. In addition, relationships between nodal properties and the severity of clinical dystonia were assessed through Spearman's correlation analyses.

**Results:**

Relative to individuals in the control group, patients with CCD exhibited decreased local nodal properties in the right globus pallidus, right middle frontal gyrus, and right superior temporal pole. The degree of centrality as well as the node efficiency of the right globus pallidus were found to be significantly correlated with ocular dystonic symptom. The node efficiency of right middle frontal gyrus was significantly related to the total motor severity. No nodal properties were significantly correlated with oral dystonic motor scores. Among CCD patients, the right hemisphere exhibited more widespread decreases in connectivity associated with the motor related brain areas, associative cortex, and limbic system, particularly in the middle frontal gyrus, globus pallidus, and cingulate gyrus.

**Conclusions:**

The assessment of morphological correlations between different areas in the brain may represent a sensitive approach for detecting alterations in brain structures and to understand the mechanistic basis for CCD at the network level. Based on the nodal properties identified in this study, the right middle frontal gyrus and globus pallidus were the most severely affected in patients with CCD. The widespread alterations in morphological connectivity, such as the cortico-cortical and cortico-subcortical networks, further support the network mechanism as a basis for CCD.

## Introduction

Idiopathic blepharospasm (BSP) is a relatively common form of adult-onset focal dystonia that can cause symptoms, such as eyelid spasms and uncontrolled blinking (1). BSP progression can, in some patients, result in the development of oromandibular dystonia known as primary craniocervical dystonia (CCD) (2), particularly within the 3-year period after BSP development (3). Despite the relatively high frequency with which this condition develops, the pathophysiology of CCD and the mechanisms whereby specific brain regions contribute to dystonic symptom are poorly understood.

Several cortical and subcortical structures have been reportedly linked to dystonia development, consistent with the classification of dystonia as a form of network disease (4). Multimodal neuroimaging methods can be used to construct models of human brain networks (5, 6), network analyses of MRI data enabling the noninvasive evaluation of brain structures in patients with dystonia. Resting-state functional MRI (rsfMRI) studies of BSP patients have revealed localized changes in brain activity levels in regions including the frontal/prefrontal cortex, cingulate, insular cortex, somatosensory regions, striatum, thalamus and cerebellum (7, 8). Functional connectivity analyses have further demonstrated reductions in connectivity between the basal ganglia and primary/secondary sensorimotor areas, the cingulate cortex, and the parietal associate cortex in BSP patients as well as decreased connectivity between cerebellum and somatosensory cortex (9). Besides, the intensity of eyelid muscle spasms in BSP patients was associated with increased BOLD activity in the sensorimotor cortex and cerebellum (10). Structural MRI analyses performed *via* a voxel-based morphometry (VBM) approach have also exhibited widespread inconsistent gray matter (GM) abnormalities in the basal ganglia and cortical areas of patients with CCD (11, 12). Most of these prior studies of GM morphometry, however, have evaluated specific regions of the brain and thus have the potential to accurately capture the complex changes in GM network connectivity supporting higher motor-related processes.

Graph theory analysis is a recently developed and powerful approach of evaluating complex connectivity within brain structures (13). Herein, we explored graph properties using a method proposed by Tijms et al. to statistically describe morphological connectivity based on intracortical similarity in individual patients with CCD based upon T1-weighted MRI scans (14). This same strategy has successfully been utilized to assess individuals suffering from Alzheimer's disease (15–17), posttraumatic stress disorder (13), chronic migraines (18), and schizophrenia (19). In comparison with blood oxygenation level-dependent (BOLD) fMRI signals, which are unable to discriminate primary or secondary functional activities in the pathological network associated with CCD. MRI morphological analyses results can directly reflect structural changes within brain regions and may offer complimentary insights regarding the pathogenesis of CCD.

## Materials and Methods

### Subjects

The present study was approved by the Institutional Review Board of Beijing Tiantan Hospital, with all patients and healthy control having provided informed consent to participate. In total, this study retrospectively analyzed 37 patients that had been clinically diagnosed with CCD and evaluated at the Functional Neurosurgery department in Beijing Tiantan Hospital between August 2018 and December 2020. These patients had presented with BSP with oromandibular dystonia, and were diagnosed with CCD by an experienced movement disorders specialist (H.M.W) and a functional neurosurgeon (K.Z). Laboratory and imaging examinations did not reveal any abnormalities, and any MRI scans exhibiting imaging artifacts were excluded from these analyses. Patients were excluded if they had a history of psychiatric disease, drug or alcohol abuse, or other secondary factors. Patients were not excluded if they had undergone prior botulinum toxin injection, as all presented with typical signs of CCD when admitted to our department. In addition to these patients with CCD, an MRI study of 30 healthy demographically and sex matched control individuals with no history of neurological disease, no family history of dystonia, and normal neuroimaging study, and neurological exam results. In addition, all participants were right-handed as determined based upon the Edinburgh Handedness Inventory.

### Clinical Assessment

Prior to MRI scanning, all patients underwent a clinical evaluation performed by an experienced movement disorders specialist (H.M.W) using the Jankovic Rating Scale (JRS) (20), Burke–Fahn–Marsden Dystonia Rating Scale (BFMDRS) (21) movement subscale, and Unified Dystonia Rating Scale (UDRS) (22). This assessment was recorded on video.

### MRI Acquisition and Preprocessing

A 3T Siemens Verio MRI scanner was used to conduct T1 structural imaging for all study participants with an 8-channel phased-array head coil. Participants had their heads stabilized using cushions, and were given earplugs. A spoiled gradient recalled sequence was used for image acquisition with the following settings: repetition time (TR) = 6.8 ms, echo time (TE) = 3.1 ms, flip angle (FA) = 12 degrees, 196 axial slices with slice thickness = 1 mm, field of view (FOV) = 24 cm × 24 cm, and data matrix = 256 × 256.

The Statistical Parametric Mapping (SPM) software (http://www.fil.ion.ucl.ac.uk/spm/software/spm12/) was used for the preprocessing of the resultant structural images. Default tissue probability maps were initially utilized to separate individual images into GM, white matter, and CSF segments, after which the DARTEL tools from SPM 12 were employed to generate a template for determining nonlinear deformations to convert images of the gray and white matter into the Montreal Neurological Institute (MNI) coordinate space. Spatial smoothing of GM data that were resampled to 2 mm^3^ voxels was conducted (Gaussian kernel with a full width at a half maximum of 6 mm) (13, 23).

### Brain Network Extraction

Intracortical similarity was used to establish GM networks for individual structural images *via* an automated data-driven approach published previously (14, 23). Briefly, network nodes were first defined 3 × 3 × 3 voxel cubes (27 voxels) in standard space, thus ensuring that the 3D cortical structures remained intact while providing a quantitative value reflective of local cortical thickness and folding. Structural similarity among cube pairs was assessed by performing correlative analyses of the 27 voxels per cube. Owing to the curvature of the neocortex, the rotation of a cube over the cortex can alter its similarity relative to a seed cube. Cubes were thus rotated to establish their maximal correlation with seed cubes over different rotations. This approach ultimately facilitated the establishment of a single-subject network for each participant. Similarity matrices were then binarized based upon whether or not similarity was significant (*p* <0.05) as established based upon false discovery rate (FDR) corrections for individual networks to facilitate unweighted and undirected graph construction.

Gray matter networks were normalized based upon the 90-node Automated Anatomical Labeling (AAL) parcellation template. Individual cubes were assigned to particular brain region of interests (ROIs) within the AAL atlas based upon the ROI to which the majority of voxels within a given cube were assigned (24). AAL ROI pairs were deemed connected with a weight (0–1) based upon the ratio of significant cube-to-cube correlations to all possible connections among all cubes within these ROIs in the previous binary network. Weighted normalized networks were then used to calculate network measurements.

### Network Properties

Network properties were determined using the GRETNA software (www.nitrc.org/projects/gretna/) (25). The sparsity (S) threshold range was from 0.10 to 0.34 with a 0.01 interval as discussed previously (13). Area under the curve (AUC) values across the S parameter range were determined for individual network metrics, generating a summarizing scalar for the topological characteristics of these networks without restricting them to a specific arbitrarily selected threshold. Global small-world metrics included: characteristic path length (Lp), clustering coefficient (Cp), normalized characteristic path length (λ), normalized clustering coefficient (γ), and small worldness (σ). Local efficiency (Eloc) and global efficiency (Eglob) were evaluated to measure the network efficiency. Nodal degree and efficiency were assessed for each AAL region.

### Statistical Analyses

Network differences between patients with CCD and controls were identified using Student's *t*-tests, with multiple comparison corrections being made using the FDR approach when comparing nodal degree and efficiency across groups. For brain regions exhibiting significant alterations (*p* < 0.05), Spearman's correlation analyses were used to assess the relationship between nodal degree or efficiency and the severity of clinical dystonia using SPSS 22 (IBM, NY, USA). With respect to inter-nodal connection comparisons, a connection matrix for each participant was created (90 × 90) according to morphological similarity and the two-sample *t*-test was used to calculate the connection difference between CCD and control group, followed by FDR corrected (*p* < 0.05), with age and gender as covariates. The analyses were completed using GRETNA toolbox.

## Results

The mean ages of the patients with CCD and control participants in this study were 54.28 ± 10.91 and 58.91 ± 8.89 years, respectively (*p* = 0.068). There were no significant differences in gender distributions between the CCD group (22 women, 15 men) and the control group (20 women, 10 men) (X2 = 0.368, *p* = 0.54). The mean age of dystonia onset in these patients was 51 ± 10.70 years, with a mean disease duration of 3.43 ± 2.59 years. The dystonia patients group includes 5 patients with isolated BSP and 32 patients with BSP and oromandibular dystonia. These patients exhibited mean BFMDRS-M and UDRS scores of 5.94 ± 3.28 and 8.05 ± 3.44, respectively. A total of 21 patients with CCD had undergone prior botulinum toxin treatment, and 20 reported an uncomfortable feeling affecting the bilateral face or eyes, such as burning sensations, soreness, undescribed sensations, dry eyes, photophobia, and eye pain.

### Global and Nodal Brain Network Alterations

No significant alterations in GM global topological properties were evident in patients with CCD, nor were there any differences between patients with CCD and controls with respect to Cp (0.1532 ± 0.0047 vs. 0.1520 ± 0.0041, *p* = 0.26), Eloc (0.1873 ± 0.0045 vs. 0.1877 ± 0.0043, *p* = 0.73), Lp (0.4870 ± 0.0097 vs. 0.4847 ± 0.0097, *p* = 0.33), Eglob (0.1205 ± 0.0023 vs. 0.1213 ± 0.0035, *p* = 0.27), γ (0.4589 ± 0.0336 vs. 0.4620 ± 0.0334, *p* = 0.71), λ (0.2705 ± 0.0033 vs. 0.2693 ± 0.0037, *p* = 0.16), or σ (0.4062 ± 0.0322 vs. 0.4097 ± 0.0320, *p* = 0.66) values.

**Figure 1:**
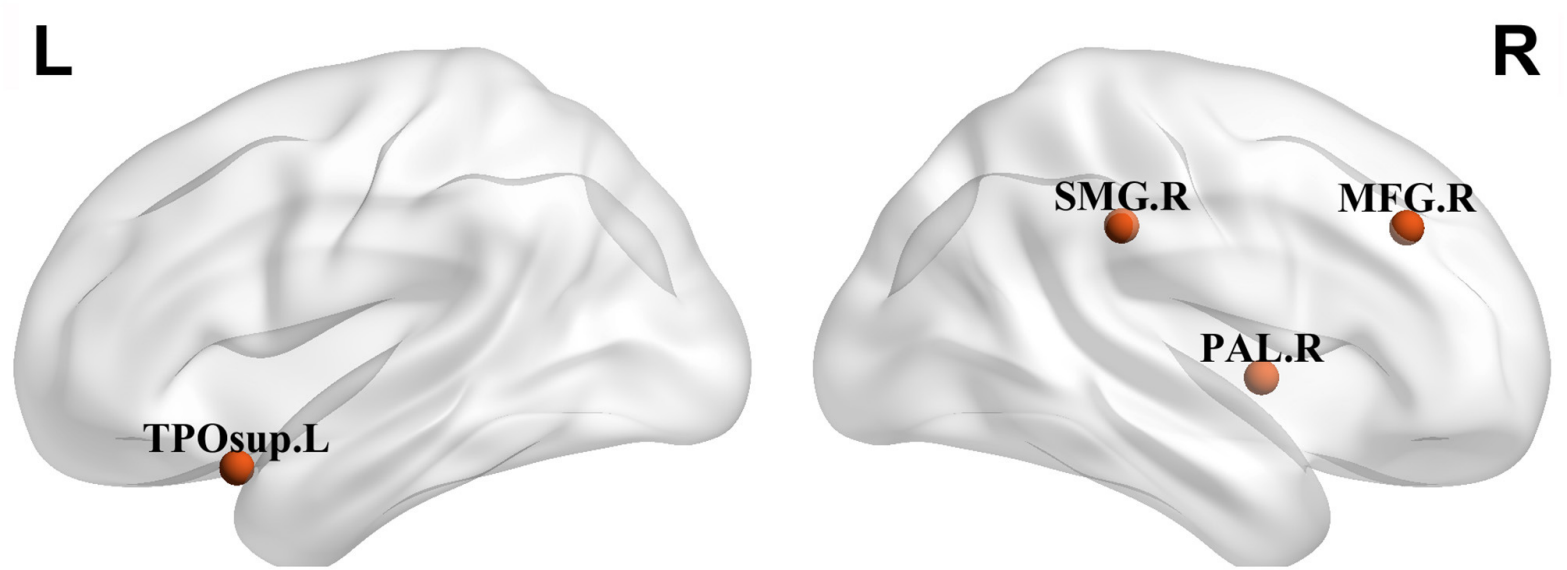
Regions with significant alterations both in nodal degree and nodal efficiency in patients with craniocervical dystonia (CCD) in comparison with healthy controls. TPOsup., temporal pole, superior temporal gyrus; MFG, middle frontal gyrus; Pall., pallidus globus; and SMG, superior marginal gyrus.

Next, we identified brain regions that exhibited significant between-group differences with respect to both nodal degree and nodal efficiency (FDR-corrected *p* < 0.05). Relative to controls, patients with CCD exhibited reduced betweenness centrality in the right middle frontal gyrus (21.5396 ± 17.4853 vs. 8.5847 ± 7.9400, *p* < 0.05) and degree of centrality in the right middle frontal gyrus (15.4150 ± 2.2661 vs. 13.2014 ± 2.9760, *p* < 0.05), right globus pallidus (9.5050 ± 1.4763 vs. 7.9703 ± 1.4763, *p* < 0.05), and left superior temporal pole (13.9392 ± 1.6284 vs. 12.5716 ± 1.6241, *p* < 0.05), together with a significantly increased degree of centrality in the right superior marginal gyrus (12.0858 ± 2.8648 vs. 14.5385 ± 2.1261, *p* < 0.05). Nodal efficiency analyses revealed that patients with CCD exhibited reductions in right middle frontal gyrus (0.2839 ± 0.0173 vs. 0.2660 ± 0.0221, *p* < 0.05), right globus pallidus (0.2367 ± 0.0174 vs. 0.2204 ± 0.0163, *p* < 0.05), and left superior temporal pole (0.2782 ± 0.0124 vs. 0.2676 ± 0.0132, *p* < 0.05) in group regions of patients with CCD patients (Figure 1).

### Correlation Between Network Alterations and CCD Severity

**Figure 2:**
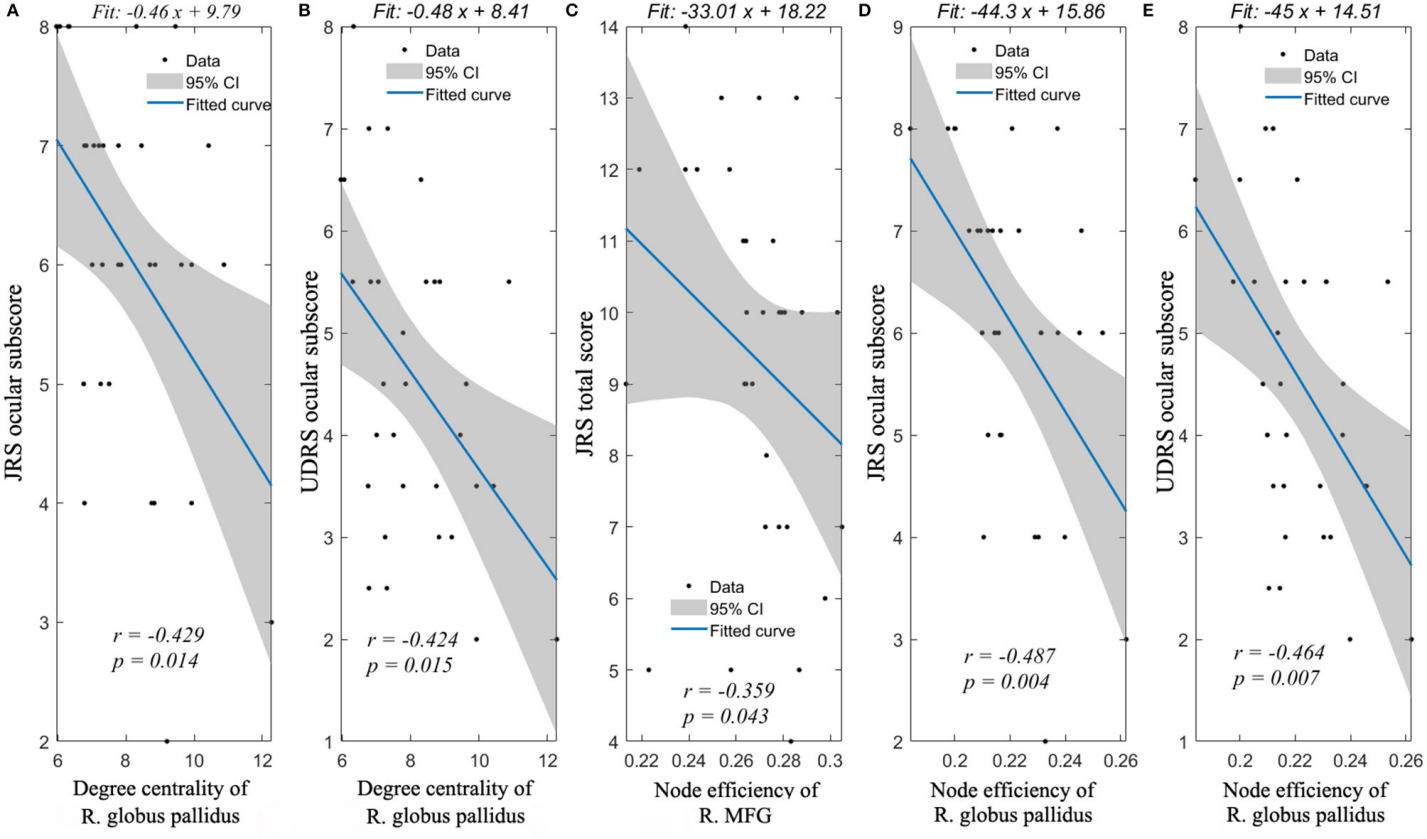
Spearman's correlation analyses between nodal properties and dystonia symptoms. Significant correlations could be found between JRS ocular subscore and degree centrality of R. globus pallidus **(A)**, UDRS ocular subscore and degree centrality of R. globus pallidus **(B)**, JRS total score and node efficiency of R. MFG **(C)**, JRS ocular subscore and node efficiency of R. globus pallidus **(D)**, UDRS ocular subscore and node efficiency of R. globus pallidus **(E)**. R., right; JRS, Jankovic Rating Scale; and UDRS, Unified Dystonia Rating Scale.

The degree of centrality of right globus pallidus was significantly correlated with JRS ocular dystonia subscore (*r* = −0.429, *p* = 0.014) and UDRS ocular subscore (*r* = −0.424, *p* = 0.015). Besides, the node efficiency of right globus pallidus was significantly correlated with JRS ocular dystonia subscore (*r* = −0.487, *p* = 0.004) and UDRS ocular subscore (*r* = −0.464, *p* = 0.007). In addition, the node efficiency of right middle frontal gyrus was significantly related to JRS total score (*r* = −0.359, *p* = 0.043) (Figure 2). No nodal properties were found to be significantly related to mouth motor scores.

### Network Connectivity Alterations

**Figure 3:**
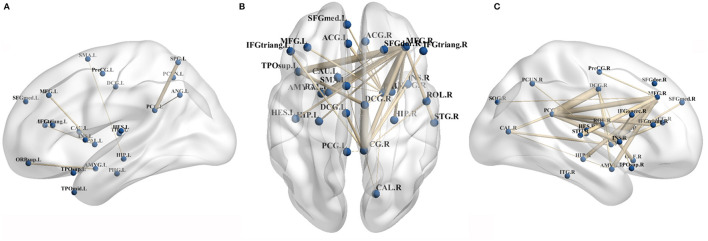
Primary CCD related decreased brain connectivity in morphological connectivity analyses. **(A,C)** Showed decreased connectivity within left and right hemisphere, respectively. **(B)** Showed decreased inter-hemispheral connectivity in patients with CCD. ACG, anterior cingulate gyrus; AMY, amygdala; ANG, angular gyrus; CAU, caudate nucleus; DCG, middle cingulate gyrus; HES, Heschl gyrus; HIP, hippocampus; IFGtriang, inferior frontal gyrus, triangular part; IFGoperc., inferior frontal gyrus, opercular part; INS, insula; ITG, inferior temporal gyrus; MFG, middle frontal gyrus; ORBsup., superior frontal gyrus, orbital part; Pall., pallidus globus; PHG, parahippocampal gyrus; PCUN, precuneus; PreCG, precentral gyrus; PCG, posterior cingulate gyrus; SFG med, superior frontal gyrus medial part; SFG dor., superior frontal gyrus dorsal part; STG, superior temporal gyrus; SMA, supplementary motor area; SOG, superior occipital gyrus; SPG, superior parietal gyrus; and TPOsup., temporal pole, superior temporal gyrus.

More widespread decreased connectivity involving the motor related areas, fronto-parietal associative cortex, and limbic system were evident in patients with CCD and the right hemisphere showed the most severely affected, particularly in the middle frontal gyrus, globus pallidus, and cingulate gyrus regions (Figure 3, Supplementary Table 1). For further details regarding the reductions in inter-hemisphere connectivity, see the Supplementary Materials (Supplementary Table 2). Several brain regions exhibited increased connectivity, as between the left superior parietal gyrus and the left posterior cingulate gyrus, the left angular gyrus and the left posterior cingulate gyrus, the right putamen and the right olfactory cortex, the left superior parietal gyrus and the right inferior frontal gyrus opercular part, and the left posterior cingulate gyrus and the right inferior temporal gyrus.

## Discussion

To date, primary regional cortical morphological measures based upon structural MRI scans, such as cortical thickness (26) or volume (12) are commonly used to study the pathological basis for CCD. In contrast, there have been few analyses of inter-regional brain morphological relationships even though they have the potential to offer valuable insights not apparent based upon local morphological measurements (27). Analyses of brain connectivity that quantify the similarity of morphological distributions between different brain regions, as were performed in this study, offer an effective approach for evaluating these inter-regional relationships. The physiological basis for such inter-regional morphological relationships can be complex. Axon tension theory posits that regions which are anatomically connected can be pulled by mechanical forces that ultimately give rise to similar morphological properties (28). Alternatively, brain regions that exhibit similar morphological distributions may be reflective of coordination between these areas in the context of development (29, 30) and learning (31, 32). Herein, we did not detect any global differences in brain organization when comparing patients with CCD to healthy controls. However, patients with CCD did exhibit certain nodal alterations, particularly in the right middle frontal gyrus and globus pallidus, with widespread changes in the morphological brain network analyses of the sensorimotor network, default mode network, and limbic system. As such, we were herein able to utilize a novel morphological brain network analyses to successfully reinforce the hypothesis that both cortical network and basal ganglia circuits are involved in the pathogenesis of CCD.

The results of this study suggest that the globus pallidus is most affected in the basal ganglia of patients with CCD, as evidenced by the detection of both nodal changes and various shifts in connectivity. Consistent with the existence of a dystonia-related cortex-basal ganglia-cerebellum circuit, globus pallidus functional and structural alterations were closely linked to the development of dystonia. A prior VBM analyses-based study found that individuals affected by focal hand dystonia, cervical dystonia, and primary generalized dystonia exhibited an increased GM volume in the globus pallidus interna (33). Diffusion tensor imaging (DTI) studies have similarly reported microstructural alterations in the white matter covering the right pallidum in patients with BSP or oromandibular dystonia (3). Furthermore, rsfMRI studies of patients with BSP have revealed them to exhibit an increased amplitude of low-frequency fluctuation (ALFF) within the pallidum (8). Together, these results all suggest that morphological alterations in the globus pallidus contribute to the altered morphological connectivity of this region. We herein further found that the degree of centrality and node efficiency of the right globus pallidus were significantly negatively correlated with BSP severity of CCD. Our structural morphological connectivity results thus provide further support for the central network node of global pallidus in dystonia.

Brain areas found to exhibit altered intrahemispheric connectivity with the globus pallidus included the opercular portion of the inferior frontal gyrus, the middle frontal gyrus, and the medial superior frontal gyrus in the right hemisphere. A majority of these areas belong to the premotor cortex or high motor processing areas, suggesting a mechanistic role for the cortical-basal ganglia connection in the development of CCD. The premotor cortex is an essential mediator of motor preparation and execution, making it central for controlling motor inhibition (34, 35). The node efficiency of the right middle frontal gyrus was particularly negatively correlated with eye motor scores in our study cohort, and the cortical eye field in the posterior middle has been suggested to contribute to the incidence of dystonic eye movements (36). In a prior task-related fMRI study, impaired ventral premotor cortex activation when whistling was specifically linked to the clinically impacted oromandibular motor system in CCD (37). Consistent with these findings, the opercular portion of the inferior frontal gyrus, which corresponds to the region of ventral premotor orofacial representation, exhibited reduced connectivity with the globus pallidus in patients with CCD in our morphological network. The inferior frontal gyrus has also been reported to be involved in regulating the blinking reflex (38). Overall, the changes in morphological connectivity with the globus pallidus in the current study provide further support for impaired motor information processing and insufficient cortical inhibition in patients with CCD.

In our morphological network analyses, we found that the most affected cortical areas within the limbic system were the cingulate gyri. In line with these findings, prior reports have found that patients with BSP exhibited increased glucose metabolism in the posterior cingulate cortex (PCC) and anterior cingulate cortex (ACC) (38). VBM analyses have revealed changes in GM volume or density within the anterior cingulate cortex in patients with BSP (39–41). The anterior cingulate gyrus is relevant for motor planning and error correction (42), while the middle cingulate cortex (MCC) is regarded as the motor area of the limbic system owing to its projection to the spinal cord, striatum, and cerebellar systems (43). Studies of rhesus monkeys have indicated that the caudal terminus of the pACC innervates the facial nucleus and is responsible for facial expression (44). In our network analyses, we found that the ACC and right MCC exhibited reduced connectivity with the globus pallidus, potentially contributing to the disruption of the facial muscle motor regulatory network in those with CCD. The amygdala reportedly exhibits significant projection to the facial region within the cingulate motor area, potentially contributing to the emotional facial expression (44). Reduced connectivity between the amygdala and the MCC indicates that this regulatory control over facial expression may be disrupted in patients with CCD. The posterior cigulate gyrus (PCG), which is a central facet of the default mode network (DMN), additionally exhibited decreased connectivity with the ACC, MCC, and insula within the limbic system, which has the potential to be associated with depression and other mood disorders that are common in patients with CCD (12). In line with our results, one prior rsfMRI study similarly detected decreases in regional spontaneous neuronal activity in the PCC using ALFF in individuals affected by BSP (8).

Together, these results support the conclusion that evaluating structural correlations between different brain regions may represent a sensitive approach in detecting the brain structural alterations and understanding the mechanistic basis for CCD symptoms at the network level. By advancing current knowledge regarding CCD-related brain connectivity, these results may guide novel approaches for predicting and/or monitoring CCD progression. However, these results are subject to certain limitations. For one, it is not clear for sure that whether the morphological abnormality is the primary or secondary due to longstanding dystonia and spatial smoothing in the preprocess of structural MRI may affect the actual morphological similarity between brain regions. Then, the morphological analyses did not include regions within the cerebellum. And the interval between the BTX injection and the MRI scan was not included in the morphological analysis, which might influence the gray matter volume. In addition, only the severity of CCD-related motor symptoms was assessed in this study, and mood disorders were not analyzed despite their clinical relevance. Correlations between the motor severity and network connectivity values were also not included in the present analyses and thus warrant future study.

## Conclusions

In these analyses, we employed a combination of MRI-based GM measurements and graph theory-based network analyses to provide evidence for altered structural network topology in patients with CCD. While CCD was not associated with any global changes in network properties, it was associated with apparent nodular changes in the right middle frontal gyrus and globus pallidus, with these alterations being significantly correlated with clinical motor severity. Morphological brain network analyses additionally highlighted changes in inter-region connectivity in widespread brain regions, such as the motor related brain areas, associative cortex, and limbic system.

## Data Availability Statement

The original contributions presented in the study are included in the article/Supplementary Material, further inquiries can be directed to the corresponding authors.

## Ethics Statement

The studies involving human participants were reviewed and approved by Institutional Review Board of Beijing Tiantan Hospital. The patients/participants provided their written informed consent to participate in this study.

## Author Contributions

XW performed acquisition of data, statistical analyses, and drafting the manuscript. WH and HW contributed to the acquisition and interpretation of data and revising the manuscript for intellectual content. DG, YL, XZ, YJ, JM, and FM responsible for the acquisition of data and revising the manuscript for intellectual content. KZ and J-gZ contributed to the study design, study supervision, and final revising the manuscript for intellectual content. All authors contributed to the article and approved the submitted version.

## Funding

This work was supported by the National Key R&D Program of China (No. 2018YFC0115401) and the National Natural Science Foundation of China (No. 81830033).

## Conflict of Interest

The authors declare that the research was conducted in the absence of any commercial or financial relationships that could be construed as a potential conflict of interest.

## Publisher's Note

All claims expressed in this article are solely those of the authors and do not necessarily represent those of their affiliated organizations, or those of the publisher, the editors and the reviewers. Any product that may be evaluated in this article, or claim that may be made by its manufacturer, is not guaranteed or endorsed by the publisher.
