# Supplementary material for: Altered Structural Brain Network Topology in Patients With Primary Craniocervical Dystonia
Source: Front Neurol. 2022 Mar 30;13:763305. doi: 10.3389/fneur.2022.763305 (PMC9005792; doi:10.3389/fneur.2022.763305)
Supplement: Supplementary file 1 [file Table_1.docx]

Supplementary Material

**Table-1.** Decreased anatomic connectivity within the same hemisphere in CCD patients relative to controls

| **Region 1** | **Region 2** | **t-score** |
| --- | --- | --- |
| Middle frontal gyrus R | Precentral gyrus R | 3.70 |
|  | Caudate nucleus R | 3.85 |
|  | Thalamus R | 3.52 |
|  | Hippocampus R | 4.21 |
|  | Amgdala R | 4.29 |
|  | Superior occipital gyrus R | 3.59 |
|  | Posterior cingulate gyrus R | 4.49 |
|  | Heschl gyrus R | 3.71 |
|  | Superior temporal gyrus R | 4.31 |
| Median cingulate and paracingulate gyri R | Amgdala R | 3.78 |
|  | Superior temporal pole R | 3.59 |
| Posterior cingulate gyrus R | Median cingulate and paracingulate gyri R | 5.13 |
|  | Anterior cingulate and paracingulate gyri R | 3.89 |
|  | Inferior frontal gyrus, triangular part R | 4.07 |
|  | Insula R | 4.29 |
|  | Rolandic operculum R | 3.95 |
| Calcarine fissure and surrounding cortex R | Posterior cingulate gyrus R | 3.90 |
|  | Amygdala R | 3.53 |
| Globus pallidus R | Anterior cingulate and paracingulate gyri R | 3.94 |
|  | Median cingulate and paracingulate gyri R | 4.04 |
|  | Inferior frontal gyrus, opercular part R | 3.72 |
|  | Middle frontal gyrus R | 5.10 |
|  | Superior frontal gyrus medial R | 3.75 |
|  | Precuneus R | 3.59 |
|  | Calcarine fissure and surrounding cortex R | 3.89 |
| Anterior cingulate and paracingulate gyri L | Globus pallidus L | 3.54 |
| Supplementary motor area L | Hippocampus L | 3.51 |
| Superior frontal gyrus, orbital part L | Amygdala L | 3.72 |
| Middle frontal gyrus L | Amygdala L | 3.76 |
