# Supplementary material for: Altered Structural Brain Network Topology in Patients With Primary Craniocervical Dystonia
Source: Front Neurol. 2022 Mar 30;13:763305. doi: 10.3389/fneur.2022.763305 (PMC9005792; doi:10.3389/fneur.2022.763305)
Supplement: Supplementary file 2 [file Table_2.docx]

Supplementary Material

**Table-2.** Decreased interhemisphere anatomic connectivity in CCD patients relative to controls

| **Region 1** | **Region 2** | **t-score** |
| --- | --- | --- |
| Precentral gyrus L | Middle frontal gyrus R | 3.70 |
| Superior frontal gyrus, dorsolateral. R | Amygdala L | 3.91 |
| Superior frontal gyrus, orbital part L | Amygdala R | 3.50 |
| Superior frontal gyrus, orbital part L | Globus pallidus R | 3.64 |
| Middle frontal gyrus L | Posterior cingulate gyrus R | 3.78 |
| Middle frontal gyrus L | Globus pallidus R | 4.10 |
| Middle frontal gyrus R | Hippocampus L | 4.07 |
| Middle frontal gyrus R | Parahippocampal gyrus L | 3.69 |
| Middle frontal gyrus R | Amygdala L | 4.47 |
| Middle frontal gyrus R | Caudate nucleus L | 3.86 |
| Middle frontal gyrus R | Globus pallidus L | 4.61 |
| Middle frontal gyrus R | Thalamus L | 3.76 |
| Middle frontal gyrus R | Heschl gyrus L | 4.10 |
| Middle frontal gyrus R | Superior temporal pole L | 4.94 |
| Inferior frontal gyrus, opercular part R | Parahippocampal gyrus L | 3.82 |
| Inferior frontal gyrus, opercular part R | Amygdala L | 3.53 |
| Inferior frontal gyrus, triangular part L | Posterior cingulate gyrus R | 4.10 |
| Inferior frontal gyrus, triangular part L | Globus pallidus R | 3.76 |
| Inferior frontal gyrus, triangular part R | Globus pallidus L | 3.61 |
| Supplementary motor area L | Posterior cingulate gyrus R | 3.86 |
| Supplementary motor area L | Amygdala R | 3.63 |
| Supplementary motor area L | Globus pallidus R | 3.88 |
| Superior frontal gyrus medial L | Amygdala R | 3.81 |
| Superior frontal gyrus medial L | Globus pallidus R | 4.02 |
| Posterior cingulate gyrus R | Insula L | 3.60 |
| Anterior cingulate and paracingulate gyri L | Globus pallidus R | 4.23 |
| Anterior cingulate and paracingulate gyri R | Globus pallidus L | 3.86 |
| Median cingulate and paracingulate gyri L | Posterior cingulate gyrus R | 4.24 |
| Median cingulate and paracingulate gyri L | Globus pallidus R | 3.69 |
| Median cingulate and paracingulate gyri R | Hippocampus L | 3.48 |
| Median cingulate and paracingulate gyri R | Parahippocampal gyrus L | 3.59 |
| Median cingulate and paracingulate gyri R | Amygdala L | 3.72 |
| Posterior cingulate gyrus R | Middle frontal gyrus L | 3.78 |
| Posterior cingulate gyrus R | Inferior frontal gyrus, triangular part L | 4.10 |
| Posterior cingulate gyrus R | Supplementary motor area L | 3.86 |
| Posterior cingulate gyrus R | Insula L | 3.60 |
| Posterior cingulate gyrus R | Median cingulate and paracingulate gyri L | 4.24 |
| Posterior cingulate gyrus R | Posterior cingulate gyrus L | 3.86 |
| Superior frontal gyrus, orbital part L | Amygdala R | 3.50 |
| Calcarine fissure and surrounding cortex R | Heschl gyrus L | 3.50 |
| Precuneus L | Globus pallidus R | 3.55 |
| Superior frontal gyrus, orbital part L | Globus pallidus R | 3.64 |
